# Primary Cervical Diffuse Large B-cell Lymphoma Incidentally Diagnosed During Evaluation of High-Grade Cervical Dysplasia: A Case Report

**DOI:** 10.7759/cureus.117551

**Published:** 2026-10-06

**Authors:** Afoma Anyadibe, Zeeshan Javed, Minh Stephenson, Olabisi Afolayan-Oloye, Anuoluwa Oyetoran, Jessica Thomas, Waqqas Tai, Ishmael Jaiyesimi

**Affiliations:** 1 Hematology and Oncology, Corewell Health William Beaumont University Hospital, Royal Oak, USA; 2 Internal Medicine, Oakland University William Beaumont School of Medicine, Rochester, USA; 3 Pathology, Corewell Health William Beaumont University Hospital, Royal Oak, USA

**Keywords:** biopsy, cervix, chemotherapy, diffuse large b-cell lymphoma, immunotherapy

## Abstract

Primary diffuse large B-cell lymphoma (DLBCL) of the uterine cervix is a rare extranodal malignancy that may mimic more common cervical pathologies. We report the case of a 33-year-old woman with human papillomavirus (HPV)-16 positivity who was found to have cervical DLBCL incidentally during evaluation and treatment of high-grade squamous intraepithelial lesion (HSIL). Histopathologic analysis following repeat excisional procedure revealed DLBCL, germinal center B-cell subtype, without residual epithelial dysplasia. Staging positron emission tomography/computed tomography (PET/CT) demonstrated fluorodeoxyglucose (FDG) uptake confined to the cervix and central uterine cavity, without nodal or distant involvement. Bone marrow and endometrial biopsies were negative. The disease was classified as stage IE extranodal DLBCL with low-risk features. The patient was treated with four cycles of R-CHOP (rituximab, cyclophosphamide, doxorubicin, vincristine, and prednisone) using a PET-adapted approach based on the Intergroup S1001 trial and achieved a complete metabolic response. This case highlights an uncommon presentation of cervical lymphoma, the importance of accurate histopathologic diagnosis, and the role of PET-adapted therapy in limited-stage disease.

## Introduction

Non-Hodgkin lymphoma accounts for approximately 4% of all malignancies in the United States, with diffuse large B-cell lymphoma (DLBCL) representing the most common subtype [1]. While extranodal involvement occurs in a significant proportion of cases, primary lymphoma of the uterine cervix is exceedingly rare, accounting for a small fraction of both cervical malignancies and extranodal lymphomas [2,3].

Primary cervical lymphoma often presents with nonspecific gynecologic symptoms, such as abnormal vaginal bleeding, pelvic pain, or a cervical mass, frequently leading to an initial suspicion of epithelial malignancy [3,4]. However, in rare instances, it may be discovered incidentally during evaluation for abnormal cytology or cervical dysplasia. The coexistence of high-grade cervical dysplasia and cervical lymphoma is particularly uncommon and may complicate the initial diagnostic evaluation

Given its rarity, there are no disease-specific prospective trials to guide management, and treatment strategies are largely extrapolated from studies of limited-stage DLBCL. Early-stage disease is highly responsive to systemic chemoimmunotherapy, and outcomes are generally favorable when appropriately managed [5].

We present a case of stage IE primary cervical DLBCL diagnosed incidentally following excisional procedures for high-grade cervical dysplasia, highlighting key diagnostic challenges and management considerations.

## Case presentation

A 33-year-old nulligravid woman with human papillomavirus (HPV)-16 positivity, prior abnormal Pap smears, and a 6.8 pack-year smoking history underwent routine cervical cancer screening. She denied abnormal vaginal bleeding, pelvic pain, or a prior diagnosis of cervical dysplasia. She also reported no constitutional symptoms, including fevers, night sweats, or unintentional weight loss.

Cytology initially demonstrated low-grade squamous intraepithelial lesion (LSIL), followed by atypical squamous cells, cannot exclude high-grade squamous intraepithelial lesion (ASC-H), with persistent HPV-16 positivity. She subsequently underwent a loop electrosurgical excision procedure (LEEP), which revealed cervical intraepithelial neoplasia III (CIN III) with positive margins. A repeat excisional procedure was performed, and histopathologic evaluation of the anterior and posterior cervical specimens demonstrated DLBCL, germinal center B-cell subtype, without residual squamous intraepithelial lesion. Immunohistochemistry showed positivity for CD20, PAX5, and BCL6 and negative staining for CD5, CD10, BCL2, BCL1/cyclin D1, and MUM1. CD30 was positive in a minor subset of cells, and the Ki-67 proliferation index was approximately 80-90%. Fluorescence in situ hybridization (FISH) was negative for MYC rearrangement, with no evidence of double-hit lymphoma (Figure 1).

**Figure 1:**
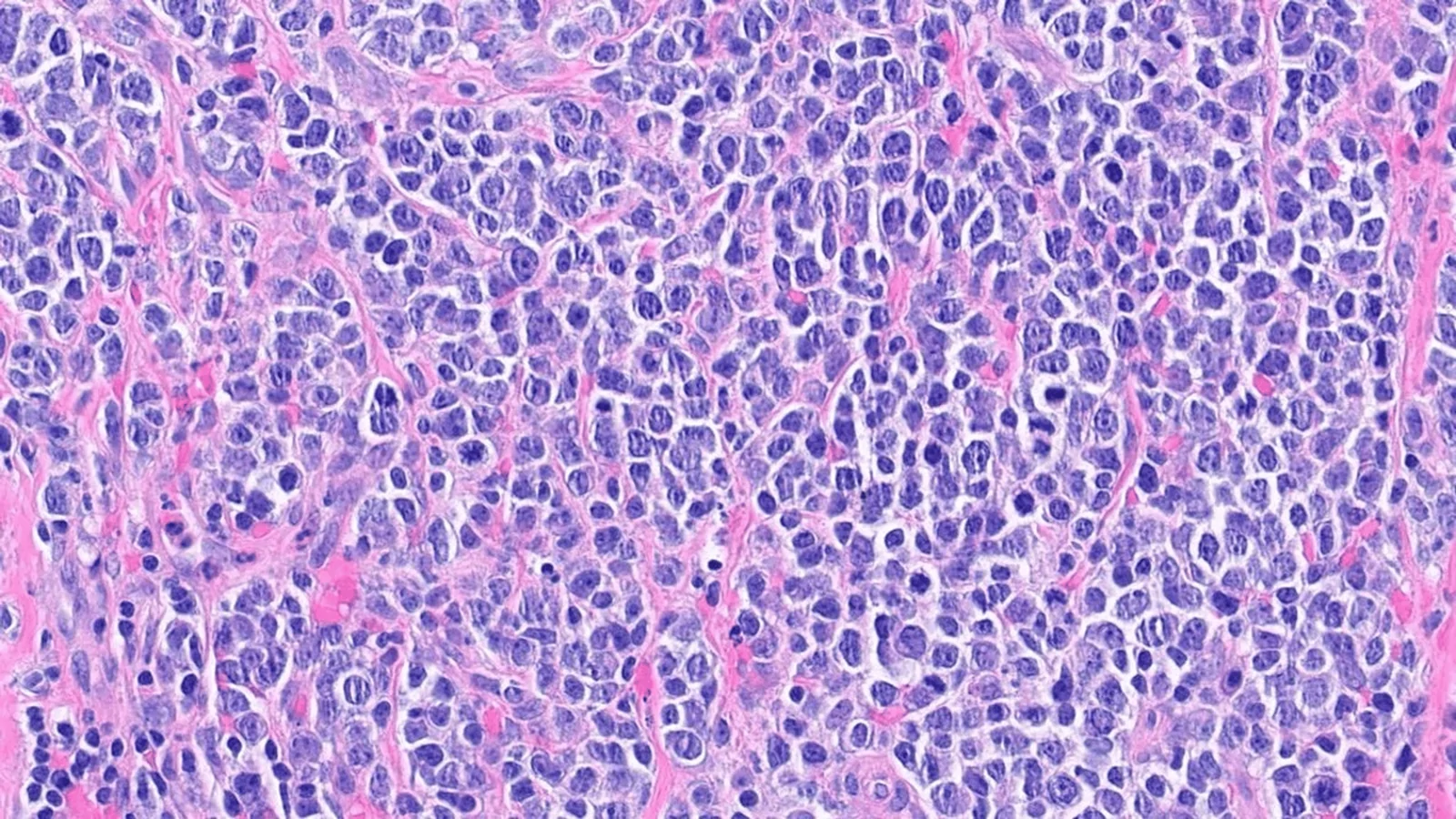
Histopathologic findings Hematoxylin and eosin (H&E) staining (40× magnification) demonstrates diffuse proliferation of large atypical lymphoid cells with prominent nuclei and scant cytoplasm, consistent with high-grade lymphoma.

Staging positron emission tomography/computed tomography (PET/CT) demonstrated fluorodeoxyglucose (FDG) uptake within the cervix and central uterine cavity, without evidence of FDG-avid pelvic or distant lymphadenopathy (Figure 2). The endometrial biopsy was negative for lymphoma, with histologically confirmed disease confined to the cervix.

**Figure 2:**
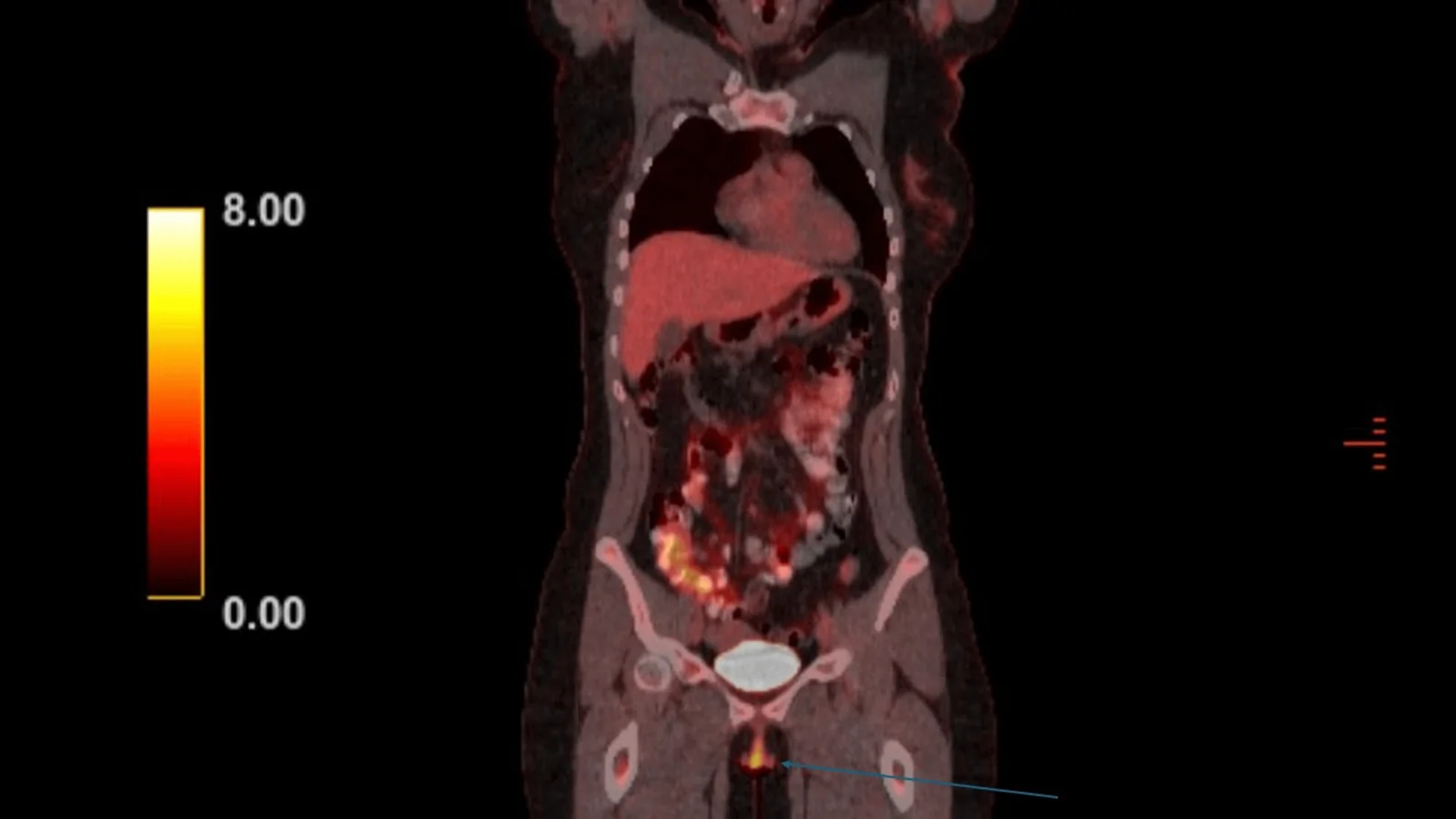
Positron emission tomography/computed tomography (PET/CT) findings Fused coronal positron PET/CT demonstrates fluorodeoxyglucose (FDG) uptake confined to the pelvis without evidence of FDG-avid nodal or distant disease. Physiologic bowel uptake is noted. The blue arrow indicates FDG uptake within the cervix.

No additional sites of abnormal FDG uptake were identified. Bone marrow biopsy showed no evidence of involvement, and endometrial biopsy was negative for lymphoma. Laboratory evaluation, including lactate dehydrogenase (LDH), was within normal limits. These findings were consistent with Ann Arbor stage IE extranodal DLBCL. She had an Eastern Cooperative Oncology Group (ECOG) performance status of 0, no bone marrow involvement, and no nodal or distant disease, corresponding to an International Prognostic Index (IPI) score of 0.

The patient was treated with R-CHOP (rituximab, cyclophosphamide, doxorubicin, vincristine, and prednisone) using a PET-adapted approach informed by the Intergroup S1001 trial. Interim PET/CT after three cycles showed no abnormal hypermetabolic lymphoma, with only treatment-related diffuse bone marrow uptake and nonspecific pelvic uptake. She completed four cycles of R-CHOP. Follow-up PET/CT demonstrated a complete metabolic response with a Deauville score of 2 and no new or progressive hypermetabolic disease. She remains on surveillance without new oncology-related symptoms.

Of note, the patient reported no desire for future fertility - a decision made prior to diagnosis.

## Discussion

DLBCL is the most common subtype of non-Hodgkin lymphoma and frequently involves extranodal sites. Primary involvement of the uterine cervix remains extremely rare, representing a small fraction of both cervical malignancies and extranodal lymphomas [2,3]. Most reported cases present with abnormal bleeding, pelvic discomfort, or a visible cervical mass [4]. In contrast, this patient was asymptomatic, and the diagnosis was made incidentally during management of high-grade cervical dysplasia. This highlights an important clinical point: lymphoma can mimic or coexist with more common gynecologic conditions, and careful histopathologic evaluation is essential when findings are atypical.

From a diagnostic standpoint, primary cervical lymphoma can be challenging because the disease often originates in the cervical stroma with relative sparing of the epithelium. As a result, cytology-based screening such as Pap smears may be non-diagnostic, and the diagnosis is frequently established only after deeper biopsy or excisional procedures. Immunohistochemical staining plays a central role in confirming the diagnosis and distinguishing lymphoma from poorly differentiated carcinoma, particularly in limited or fragmented specimens.

Staging evaluation is critical, as it directly impacts management. In this case, FDG-PET imaging demonstrated disease confined to the cervix without nodal or distant involvement, consistent with stage IE disease. Despite this localized appearance, DLBCL is considered a systemic disease. Local excision alone is insufficient due to the risk of occult microscopic dissemination, and systemic chemoimmunotherapy remains the cornerstone of treatment even in limited-stage presentations [5].

Recent prospective data support response-adapted strategies in this setting. The Intergroup S1001 trial demonstrated that patients with non-bulky stages I-II disease who achieve a negative interim PET after three cycles of R-CHOP can be treated with a total of four cycles without routine consolidative radiation, with excellent long-term outcomes [6].

Fertility considerations are an important component of care in younger patients receiving cyclophosphamide-containing regimens. Although this patient did not desire future fertility, early counseling regarding fertility preservation should be offered when appropriate [7].

Guideline-based management remains central to treatment planning. Current National Comprehensive Cancer Network (NCCN) guidelines recommend systemic chemoimmunotherapy for limited-stage DLBCL, with increasing incorporation of PET-adapted strategies to individualize treatment intensity [8].

Population-level analyses further support favorable outcomes in primary lymphomas of the female genital tract when treated with systemic therapy. A large retrospective study demonstrated that patients with localized disease have excellent survival outcomes, reinforcing that these tumors behave similarly to nodal DLBCL when managed according to standard lymphoma protocols [9].

This patient had several favorable prognostic features, including early-stage disease, normal LDH, and good performance status. Outcomes in this group are excellent, with long-term survival rates exceeding 90% in the rituximab era. The addition of rituximab to CHOP-based regimens has significantly improved both progression-free and overall survival and remains the standard of care [10]. The goal of therapy in such cases is to preserve these high cure rates while minimizing long-term toxicity.

The coexistence of cervical dysplasia and lymphoma in this case was likely coincidental, as there is no established evidence that HPV-associated dysplasia contributes to the development of cervical DLBCL. However, having both processes present in the cervix can complicate the diagnosis, particularly when only small or superficial tissue samples are available. Careful pathologic evaluation is therefore important to distinguish lymphoma from an epithelial malignancy and avoid unnecessary surgical treatment

Overall, this case highlights the importance of maintaining a broad differential diagnosis in atypical cervical pathology, reinforces the role of immunohistochemistry in diagnosis, and supports treating even localized disease as a systemic lymphoma. With appropriate therapy, early-stage primary cervical DLBCL carries an excellent prognosis.

## Conclusions

Primary cervical DLBCL is a rare entity that may be identified incidentally during evaluation of cervical dysplasia, underscoring the importance of careful pathologic assessment when findings are not consistent with typical gynecologic malignancies. Early recognition is essential to avoid misdiagnosis and unnecessary surgical intervention. Although the disease may appear localized on imaging, it should be approached as a systemic lymphoma requiring systemic chemoimmunotherapy. Patients with limited-stage disease and favorable risk features generally have excellent outcomes in the modern treatment era, particularly with response-adapted strategies. This case highlights the need to maintain a broad differential diagnosis in atypical cervical pathology and reinforces that timely, guideline-directed management can achieve high cure rates while minimizing morbidity.
